# Caloric test and video head impulse test sensitivity as vestibular impairment predictors before cochlear implant surgery

**DOI:** 10.6061/clinics/2019/e786

**Published:** 2019-03-04

**Authors:** Roseli Saraiva Moreira Bittar, Eduardo Setsuo Sato, Douglas Josimo Silva-Ribeiro, Jeanne Oiticica, Raquel Mezzalira, Robinson Koji Tsuji, Ricardo Ferreira Bento

**Affiliations:** I Departamento de Otorrinolaringologia, Faculdade de Medicina FMUSP, Universidade de Sao Paulo, Sao Paulo, SP, BR; II Departamento de Otorrinolaringologia, Universidade Estadual de Campinas, Campinas, SP, BR

**Keywords:** Caloric Test, Video Head Impulse Test, Vestibular Ocular Reflex, Cochlear Implant

## Abstract

**OBJECTIVES:**

Currently, cochlear implant procedures are becoming increasingly broad and have greatly expanded. Bilateral cochlear implants and cochlear implants are more frequently applied in children. Our hypothesis is that the video head impulse test may be more sensitive than the caloric test in detecting abnormal vestibular function before cochlear implant surgery. The objective of this study was to compare the video head impulse test and caloric test results of patients selected for cochlear implant procedures before surgery.

**METHODS:**

The patients selected for cochlear implant surgery were submitted to a bithermal caloric test and video head impulse test.

**RESULTS:**

By comparing angular slow phase velocity values below 5° in the bithermal caloric test (hypofunction) and video head impulse test with a gain lower than 0.8, we identified 37 (64.9%) patients with vestibular hypofunction or canal paresis and 21 (36.8%) patients with abnormal video head impulse test gain before the cochlear implant procedure. Of the 37 patients with caloric test vestibular hypofunction, 20 (54%) patients exhibited an abnormal gain in the video head impulse test.

**CONCLUSION:**

The caloric test is more sensitive than the video head impulse test (Fisher's exact test, *p*=0.0002) in detecting the impaired ear before cochlear implant delivery. The proportion of caloric test/video head impulse test positive identification of abnormal vestibular function or caloric test/video head impulse test sensitivity was 1.8:1.

## INTRODUCTION

Cochlear implants (CI) were first developed over 30 years ago to achieve hearing rehabilitation in patients with profound sensorineural hearing loss. Some patients have a tendency to also present vestibular disorders since a common aetiology could target both systems 1. Usually, approximately 50% of CI recipients present abnormal vestibular function before the procedure 2-4. In addition, the CI procedure could lead to vestibular function impairment because its mechanism of neural electrical stimulation may be potentially harmful to the vestibular end-organs 5.

The vestibular system is one of the main input channels that settles dynamics behaviour mainly through ocular and postural reflexes. One of the vestibular system's main functions is to evoke compensatory eye movements during head motion to achieve gaze stabilization. Gaze stabilization enables a stable visual environment perception while walking or running. Gaze stabilization is mainly elicited by the angular vestíbulo-ocular reflex (aVOR), which is one of the key functions of the semicircular canals 6.

Until recently, the CI side was chosen based exclusively on audiological characteristics and/or anatomical aspects. However, considering the increasing reports of vertigo, dizziness and imbalance after CI, vestibular function may be another aspect to consider before CI side embracement 7. The goal is to minimize postoperative CI complications by choosing whenever possible the side with the worse vestibular function. The problem is especially critical with children patients since vestibular input is crucial for healthy sensorial development and spatial perception. Since bilateral vestibular hypofunction may lead to prolonged balance impairment, vestibular function saving is particularly important when preoperative vestibular hypofunction is observed or when bilateral CI is planned 2.

The caloric test (CT) is the most widely used test to evaluate vestibular disorders. The CT measures vestibular function at very low frequencies (approximately 0.002-0.004 Hz) by bithermal caloric stimulation of the lateral semicircular canal. The limitations of this test are well known 8 because in everyday life, head movements occur at higher frequencies and involve three-dimensional spatial planes 9. The angular slow phase velocity (ASPV) is a quantitative variable used to determine the presence of vestibular paresis or hypofunction. According to the literature, after CI, a mean 10^°^/second reduction in ASPV is observed during the CT of the implanted ear compared with a mean of 1^°^/second at the contralateral ear 2. At our department, we have previously observed and reported a 20% impairment after CI in the implanted ear 10. The potential reasons for this decline after surgery may include progressive postoperative inner ear change due to fibrosis, which might delay the endolymphatic flow in the semicircular canal; immediate damage due to surgical trauma, which might affect the function of the vestibular system; or a thermal spread decline during the caloric test due to anatomical structural changes 2.

The video head impulse test (vHIT) records and quantifies high-frequency vestibular function with passive, small-amplitude and high-acceleration head rotations around the main three-dimensional spatial planes (yaw, roll, and pitch). The vHIT appears to be a very good screening test for verifying a VOR gain and determining the semicircular canal function. Gain refers to the eye movement magnitude that accompanies the head impulse. If the vestibulo-ocular reflex (VOR) is normal, the patient is able to maintain his/her gaze on the target, and the eye movement displays the same angular velocity in the same plane and in the direction opposite to the head movement. In this case, the gain is equal to 1. In cases of unilateral vestibular paresis or hypofunction, the vHIT usually shows a reduced ipsilateral gain with compensatory corrective saccades, which could be overt or covert. When the corrective saccade occurs after the head movement has stopped, the saccade is called an *overt* saccade. An overt saccade indicates an unsettled VOR due to impairment in the stimulated canal. During vestibular compensation, this saccade could be replaced by another saccade that occurs during head movement, i.e., the so-called *covert* saccade. An abnormal vHIT is highly suggestive of a peripheral vestibular lesion. vHIT appears to be useful since it provides additional and complementary information about the function of the anterior and posterior canals 6,11.

At our department, the preoperative vestibular assessments of adult patients undergoing CI include the CT in addition to other methods as previously described 12. The use of vHIT was recently incorporated into our service routine since it is important for vestibular evaluations. However, the following question still remains unanswered: could vHIT replace the bithermal CT for vestibular impairment diagnosis before CI delivery? Several studies have compared vHIT and CT outcomes among patients with vestibular complaints. According to the literature, vHIT could not replace the CT as both tests assess different VOR features 9,13,14.

A possible explanation for the dissonant findings between both tests is the complex anatomic and physiologic VOR network. The sensory organ that perceives angular head acceleration and deceleration is the so-called crista ampullaris, which gives rise to the angular VOR and contains cytoarchitecture type I and type II hair cells. The crista ampullaris receives regular and irregular neural afferent discharges. The type I cells are at the middle of the crista ampullaris and decode high-frequency and fast head movement accelerations. These cells are connected to irregular afferent neural fibres. In contrast, the type II cells are located at the periphery of the crista and decode low-frequency and acceleration head movements. These cells are connected to regular afferent neural fibres. Our previous experience with patients suffering from dizziness in our neurotology practice suggests that the sensitivity of the CT and vHIT in detecting vestibular impairment was at a 3:1 proportion 15.

Despite our previous results, our hypothesis is that in contrast to our regular observations among chronic vestibular impairment patients, in the specific case of CI recipients, vHIT may be more sensitive than the CT. Our expectation is that vHIT could be a good screening and predictor test of the best ear for CI since some patients already present labyrinth impairment prior to the CI procedure. To achieve this goal, we evaluated patients before the CI procedure using both the CT and vHIT at the same cross-section time and compared the results.

## MATERIALS AND METHODS

### Study design

This study adopted a cross-sectional cohort design previously approved by the Ethics Committee of the Clinical Hospital University of Sao Paulo School of Medicine (N^0^. 0983-07) and complies with the Helsinki Declaration of 1975.

### Study population

Our sample comprised 57 subjects, including 33 females and 24 males with a mean age and standard deviation of 49.6±15.3 years. The selected patients underwent CI at the Surgical Neuro Otology Section of the Otorhinolaryngology Department at our institution. The participants were subjected to a clinical neurotology evaluation before the CI procedure between January and December 2015.

### Procedures

The clinical complaints were record before the CI procedure. All patients were subjected to a standard clinical evaluation protocol including a medical history, ENT and cranial nerves examination, static and dynamic balance tests (Romberg and Fukuda), and coordination tests (diadochokinesis with alternating pronation and supination of the forearms and index-nose test). The participants performed the bithermal CT and vHIT on the same day before the CI procedure.

The CT was carried out with water at 30^°^ and 44^°^ Celsius for 40 seconds. Each external auditory canal was irrigated separately with a 5-minute interval between the irrigations. Interacoustics VN415 and ICS Charts 200 Otometrics were used as devices for the bithermal CT. ASPV values under 5^°^/second were considered to indicate vestibular hypofunction in each ear, and ASPV values equal to 0^°^/second were considered to indicate canal paresis 16-18.

**Figure 1:**
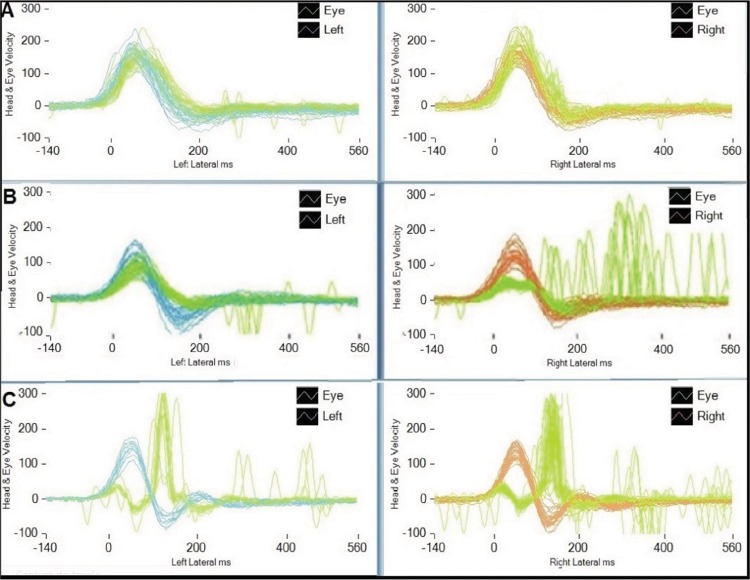
vHIT chart: (A) normal test, (B) low gain and refixation overt saccades on the right side, (C) bilateral vestibular loss (ICS Impulse Otometrics).

Eye See Cam vHITInteracoustics and ICS Impulse Otometrics were used as the devices for vHIT. The horizontal VOR was evaluated. Small-amplitude, high-acceleration and passive head rotations were applied around the horizontal plane (yaw) at approximately 20^°^ with a mean velocity of 150^°^/second, mean acceleration of 1000 to 2500^°^/second^2^ and the patients' gaze on a target placed 1 metre in front of him/her. At least 20 suitable head rotational impulses to the right and left were recorded during each test. The test was considered abnormal if the VOR gain was lower than 0.8 (Figure 1). 9,19,20.

### Statistical analysis

The subjects' mean age and standard deviation were calculated and described. To analyse the clinical data, Fisher's exact test was used. The statistical significance was set at α=0.05.

## RESULTS

Our study sample included 57 subjects (114 ears), including 33 females and 24 males with a mean age and standard deviation of 49.6±15.3 years. The deafness aetiology distribution is shown in Table 1.

The 114 ears were individually evaluated. Thirty-seven abnormal caloric tests and 21 abnormal vHIT tests were found among our 114-ear sample. We considered ASPV absolute values below 5° on the bithermal CT and a gain lower than 0.8 on the vHIT abnormal. The CT was more sensitive than vHIT (*p*=0.0002). The data are shown in Table 2 and were derived by applying a 2x2 bicaudal Fisheŕs exact test. By comparing the abnormal results or sensitivity in detecting vestibular impairment between the CT and vHIT, the proportion was 1.8:1. Therefore, we identified nearly 2 abnormal CT to each abnormal vHIT.

## DISCUSSION

CI are currently the standard hearing rehabilitation procedure for patients with profound hearing loss. CI were developed to restore hearing function; however, damage to vestibular function should be considered since the systems are related and share mutual neural network connections. Since bilateral vestibular hypofunction usually accounts for remarkable disability, knowledge of the pre-CI vestibular function is pivotal 2. Prior knowledge of the vestibular status enhances the diagnostic index, prevents iatrogenic damages and helps the management of possible post-operative vestibular disabilities 12. In cases where no other information is available for choosing the CI ear, the side with the worst vestibular function should be chosen for the procedure 21.

Vestibular disability occurs not only in patients who had a normal balance status before the CI but also in those with preoperative vestibular hypofunction. It is well known that residual vestibular function is essential for postural control in patients with vestibular impairment. Therefore, maintaining residual vestibular function is crucial for minimize balancing disabilities after the CI procedure 1.

Vestibular paresis is associated with a poor prognosis and severe restrictions in daily life activities, such as walking in low-lit environments or on uneven ground, swimming, driving fast, etc. A frequent complaint in these cases is oscillopsia during head movements mainly in the dark 22. The worst post-operative scenario is bilateral vestibular paresis (BVP) since vestibular rehabilitation is limited, and balance improvement does not exceed 50% in these cases 23.

No vestibular test is sensitive enough to be recommended as a single test. Ideally, all five vestibular sensors should be tested 24. Our routine includes a six-step evaluation before surgery to recognize harm and manage any vestibular impairment. In our clinical practice, 72% of the candidates selected for the IC procedure display unilateral or bilateral vestibular impairments 12. We preferentially adopted objective measurements to determine the horizontal canal status because of its remarkable sensitivity in recognizing vestibular impairments. In our experience with CI preoperative evaluations, the clinical head impulse test (cHIT) is sensitive enough to detect severe vestibular impairment confirmed by the caloric test 12.

The vHIT has better resources than the cHIT and is expected to provide more information about the VOR 19. In vestibular loss, there is VOR impairment with gain reduction and the presence of refixation saccades, which can be unnoticeable (catch-up covert saccades) or visible (catch-up overt saccades). cHIT is able to detect the overt saccades, while the covert saccades remain unnoticeable 25,26. We did not consider isolated presence of saccades but the low gain, since saccades frequency may increase with age even with normal gain 27,28.

Our experience with dizziness at our clinical practice is that the sensitivity in detecting vestibular impairment between the CT and vHIT was 3:1 15. Our hypothesis was that in contrast to our observations with chronic vestibular diseases vHIT could be proportionally more sensitive in specific cases, like CI recipients'. By comparing the results between the two tests, the sensitivity rate of CT/vHIT in vestibular disability detection was 1.8:1. Our results suggest that vHIT is a good predictor but does not replace the CT 29.

## CONCLUSION

In our study sample of cochlear implant recipients, the caloric test was more sensitive in detecting vestibular disability than vHIT at a rate of 1.8:1.

## AUTHOR CONTRIBUTIONS

Bittar RS was responsible for the project drafting, construction and review. Sato ES and Silva-Ribeiro DJ were responsible for the data collection. Oiticica J and Mezzalira R were responsible for the manuscript preparation and revision. Tsuji RK was responsible for the surgical procedures and clinical follow-up. Bento RF was responsible for the surgical procedures.

## Figures and Tables

**Table 1:** Deafness aetiologies recorded in the sample before the CI procedure.

Idiopathic	27	Menierés Syndrome	1
Meningitis	8	Genetics	1
Otosclerosis	7	Wolfraḿs Syndrome	1
Head Trauma	3	Hyperbilirubinemia	1
Chronic otitis media	2	Ototoxicity	1
Rubella	2	Jugular Glomus	1
Virus (non-rubella)	2		

**Table 2:** Results indicative of hypofunction or paresis on the CT and abnormal vHIT gain in our 114-ear sample before the CI procedure. The signal (*) indicates statistical significance.

Patients/tests	Hypofunction or paresis on CT	Absence of hypofunction or paresis on CT	
normal vHIT	17	19	36
abnormal vHIT	20	1	21
	37	20	*p*=0.0002*
